# The correlation between surgical, anaesthetic and obstetric workforce and surgical volume: a modelling study

**DOI:** 10.3389/frhs.2026.1920090

**Published:** 2026-10-08

**Authors:** Yuki Julius Ng, Alex J. van Duinen, Håkon A. Bolkan, Anita Gadgil, Priti Patil, Nobhojit Roy

**Affiliations:** 1 Department of General Surgery, University Hospital Bristol and Weston Trust, Weston-Super-Mare, United Kingdom; 2 Department of Public Health and Nursing, Norwegian University of Science and Technology, Trondheim, Norway; 3 Clinic of Surgery, Trondheim University Hospital St Olav’s Hospital, Trondheim, Norway; 4 WHO Collaborating Centre for Emergency, Critical and Operative Care, The George Institute for Global Health, New Delhi, India; 5 Department of Statistics, BARC Hospital, Mumbai, India; 6 Department of Global Public Health, Karolinska Institutet, Stockholm, Sweden; 7 Centre for Leadership in Global Health, University of Global Health Equity, Butaro, Rwanda

**Keywords:** global surgery, surgeon anaesthetic and obstetric workforce, surgical policy, surgical volume, workforce policy

## Abstract

**Introduction:**

The Lancet Commission on Global Surgery has stated that two-thirds of the world's population does not have adequate access to surgical and anaesthesia care. Surgical, Obstetric and Anaesthetic (SAO) workforce and surgical volume (SV) are two of the six indicators recommended for monitoring and planning. This study aims to examine the correlation between SAO density and SV at the national level.

**Methodology:**

A cross-sectional ecological regression study was conducted with SAO workforce per 100,000 population and SV per 100,000 population, extracted from the World Bank Database. Countries that reported both indicators were included. If SAO was not available for the corresponding SV year, it was adjusted according to the population growth. A logarithmic regression model was used to describe the nonlinear association between SAO workforce density and surgical volume with SPSS. Model fit (R^2^) analysis, diagnostics, and a model implied marginal slope analysis were conducted.

**Result:**

A total of 57 countries were included, with 37 (64.9%) requiring adjustment. The fitted logarithmic regression model (y = 884 ln(x) + 685) had an R^2^ of 0.524. At an SAO density of 10 per 100,000 population, the fitted model predicted approximately 2720 procedures per 100,000 population. At 20 and 40 SAO, 3333.7 (95% CI for fitted mean: 2917.2–3750.2) and 3946.5 (95% CI for fitted mean: 3470.7–4,422.3) procedures are expected, respectively. The marginal slope analysis of our model was progressively smaller at higher SAO densities.

**Conclusion:**

This study provides empirical insights into the nonlinear relationship between SAO workforce density and surgical volume. The findings highlight the need to balance workforce expansion with wider health-system capacity and may contribute to the future development of more informative surgical planning and performance indicators.

## Background

The Lancet Commission on Global Surgery (LCoGS) has estimated that 5 billion out of 6.9 billion people in 2015, did not have access to safe and affordable surgical and anaesthesia care when needed (1). An estimated 313 million procedures are performed globally per annum whilst only 6% are performed in the poorest nations, where they are the most populous countries globally (1). An estimate of a further 321 million surgical procedures were in lack globally (2).

The LCoGS described six goals to be achieved by 2030, including the number of specialist surgeons, anaesthesiologists, and obstetricians (SAO), namely 1.1, 0.6, and 0.5 million, respectively (3). Only 20% of the global SAO workforce is working in the Low-, and Lower Middle-Income Countries (LLMIC) (1). Holmer et al. plotted the SAO workforce against maternal survival rates, and a regression line was fitted showing a correlation between an increase of SAO density and improved maternal survival (3). A steep increase in maternal survival was found between 0 and 20 SAO per 100,000 population, and a relatively less steep improvement with 40 SAO per 100,000 population. These thresholds of 20 and 40 SAO per 100,000 population corresponded with the estimated Surgical Volumes (SV) of 2,917 and 5,834 procedures per 100,000 population, respectively (4). Based on this, the LCoGS decided to aim for a minimum of 20 SAOs per 100,000 population as an interim goal by 2030 as this would provide missing procedures globally (1).

To estimate the SV threshold, the LCoGS used three different strategies. The first strategy was to assess surgical rates plotted against life expectancy; at 74–75 years old life expectancy, 4,392 procedures per 100,000 population was estimated. The second investigated SV in relation to maternal mortality ratio aiming for a ratio below 100; resulting in an estimated SV of 5,028 operations per 100,000 population. The third strategy estimated the need for surgery according to the global burden of disease within regions, and this strategy estimated a minimum SV of 4,723 per 100,000 population. These strategies has identified a narrow range of surgical procedures as indicators which concluded to be 5,000 procedures per 100,000 population (5). The calculations done by the Lancet Commission on surgical workforce and volume have guided the benchmarks for 2 of 6 the indicators, 20 SAO density by 2030 and the Surgical Volume of 5,000 per 100,000 population (1). However, these benchmarks were calculated with the assumption that productivity does not change with a change in workforce. Therefore, the L benchmark of 20 SAO by 2030 might not necessarily show that 5,000 surgical procedures can be achieved by 2030. Most LLMICs have an SV below the LCoGS target of 5,000 procedures per 100,000 population and SV has a positive association with Gross Domestic Product (6). Also, many procedures globally are performed by non-specialist physicians, and in some parts of the world specially train non-physician healthcare staff to task shift for anaesthesia care (7, 8). In addition, in many countries surgical residents perform many of the more basic operations such as gallbladder removals and hernia surgery (9). Therefore assessing only the specialist workforce results in an underestimation of the overall surgical, obstetric and anaesthesia workforce. The aim of this study is to assess the correlation between surgical density and SV, to identify the required productivity to reach the SV benchmark.

## Methodology

This is a cross-sectional ecological regression study used available reported global data from the World Bank Database on the number of specialist surgeons, anaesthesiologists and obstetrician density per 100,000 population and compared it with the SV per 100,000 population (10, 11). The data spans over 12 years from 2011.

The latest number of specialist densities and the SV were included if reported in the same year. Countries reporting more than 10,000 surgical procedures per 100,000 population were classified as high volume observations and excluded from the conservative policy focussed regression model. This fixed operational threshold excluded 10 countries: Australia, Austria, Belgium, Brazil, Colombia, Denmark, Estonia, Finland, Latvia and Sweden. The threshold was transparent and reproducible but was not intended as a formal statistical definition of an outlier. As a secondary unrestricted analysis, the regression was repeated using all 67 countries with valid SAO and SV data to examine the influence of very high-volume health systems on the fitted relationship.

Given the cross-sectional nature of the data, the relationship between SAO workforce density and SV is interpreted as an association rather than a causal effect. The observed relationship reflects the combined influence of workforce availability and broader health system capacity, including infrastructure, operating theatre availability, and service organisation, which were not directly measured in this study. Therefore, this model should be understood as an empirical description of the association between workforce density and surgical output at the system level. The correlation between SAO density and SV was visualised using a scatter plot. The SAO workforce and SV were both reported as per 100,000 population.

Before prioritising the most recent eligible SV estimates, 31 countries had SAO workforce density and SV reported corresponding year. In eight of these countries, a more recent eligible SV estimate was available and was prioritised for the updated dataset; the corresponding SAO density was therefore adjusted to the newer SV reporting year. Consequently, the unrestricted 67 country dataset comprised 23 exact corresponding year observed and 44 were temporally adjusted. Application of the primary policy focussed threshold of <10,000 SV per 100,000 population excluded 10 high volume observations, of which three were exact corresponding year observations and seven were temporally adjusted. The final 57 country primary model therefore comprised 20 (35.1%) exact corresponding year and 37 (64.9%) temporally adjusted observations. Countries with non-corresponding reported years were included after adjusting the SAO workforce according to population growth with the following formula.

$$\mathrm{SAO}\;\mathrm{in}\;\mathrm{year}\;Y\;=\;\mathrm{SAO}\;\mathrm{in}\;\mathrm{year}\;X\;*\left(\frac{\;population\;in\;year\;x}{\;population\;year\;y}\right)$$

The correlation between the lower and upper SAO indicators with the number of procedures as described in the LCoGS was studied. Several conventional functional forms, including linear, logarithmic, power, exponential and polynomial relationships, were explored. The logarithmic model was used for the main analysis as an interpretable descriptive representation of the observed nonlinear association between SAO workforce density and surgical volume, characterised by progressively smaller model-implied increases in surgical volume at higher SAO densities. The model was intended to describe the association over the observed data range and not to represent a finite saturation point or maximum surgical capacity. To calculate the formula of the regression model, we used the following equation:

y=a⋅ln(x)+b

Where:

y is the dependent variable, in this case, SV per 100,000 population.
x is the independent variable, in this case, SAO per 100,000 population.
a is the coefficient representing the slope. Which was calculated with the formula = Slope(Volume, ln(x))
b is the y-intercept when ln(x) = 0 or x = 1. Which was calculated with the formula = Intercept(Volume, ln(x)). ln(x) is the natural logarithm of x.

IBM Statistical Package for the Social Sciences(SPSS) version 22 was used for analysis and visualization. The country was the unit of analysis, with each country contributing equally to the regression; the model therefore estimates a country-level rather than population-weighted association. A linear regression was performed to find the best-fit line for the data points of (ln(x), y). The regression coefficients a and b were calculated by minimizing the sum of the squared difference between the observed y-values and the values predicted by the model. The best fit of the regression model was determined with R-squared (R^2^) on SPSS. Predicted surgical volumes were calculated from the fitted logarithmic regression model. Uncertainty around the estimated mean relationship was represented using 95% confidence intervals for the fitted mean response. As individual countries were also described relative to the fitted relationship, 95% individual prediction intervals were calculated to account for residual variation between country observations. Both intervals were generated from the regression model in SPSS using a 95% confidence level and the appropriate t distribution with 55 residual degrees of freedom. Regression coefficients were reported together with standard errors, 95% confidence intervals, and *p*-values.

A corresponding year model was also fitted using the 31 countries for which SAO workforce and SV data were available for the same reporting year. This model was compared with the primary model, which prioritised the most recent eligible SV estimates and used population adjustment where reporting years differed. The comparison between the models is presented in Supplementary Table S2.

### Regression diagnostics

Model assumptions were assessed through examination of standardised residual plots, normal probability plots and Cook's distance statistics to identify potential heteroskedasticity and influential observations. These diagnostic measures were used to assess model fit and influence but were not used as automatic exclusion criteria.

### Model implied marginal slope analysis

For the fitted logarithmic relationship y = aln(x)+b, the model-implied marginal slope was calculated using the first derivative:

$\frac{dy}{dx}=\frac{a}{x}$, where (a) = regression coefficient

here a is the fitted regression coefficient for ln(SAO) and x represents SAO workforce density per 100,000 population. The model-implied marginal slope describes the local slope of the fitted logarithmic curve at a given SAO density and therefore represents the model-predicted difference in surgical volume associated with a one-unit difference in SAO density at that point on the curve. This quantity is a mathematical property of the fitted logarithmic model and was not interpreted as a sensitivity analysis of the study assumptions or as a causal effect of increasing the SAO workforce.

The model-implied marginal slope was calculated across SAO densities from >0 to 40 per 100,000 population to illustrate how the fitted association varied across workforce densities, including the SAO reference values discussed by the LCoGS. As the logarithmic model and its derivative are undefined at SAO = 0, no estimate was calculated at zero. These estimates are inherently dependent on the selected logarithmic functional form and should be interpreted as descriptive properties of the fitted cross-country relationship rather than as estimates of the causal effect of adding SAO specialists.

### Ethical considerations

Ethical approval was not required as this study contains only secondary data analysis of a publicly available dataset provided by the World Bank.

## Results

In total, 57 countries were identified that reported both SAO density and SV. Of these countries, 20 (35.1%) were included with corresponding year data and 37 (64.9%) were included with year-adjusted data (Table 1). Both corresponding and year-adjusted data were included in the scatter plot and a logarithmic regression model demonstrated a significant association between SAO workforce density and surgical volume (*β* = 884.12, SE = 113.536, 95% CI: 656.6–1,111.7, *p* < 0.001). The regression model was derived from calculating the coefficients, SV = 884  ×  ln(SAO) + 685 explained 52.4% of the variance in surgical volume across countries R^2^ = 0.524 and the Adjusted R^2^ = 0.516 [Figure 1]. The diagnostics of this regression model with residual statistics were centred around zero, indicating no systematic bias in model predictions with a maximum standardized residual of 3.514. The maximum Cook's distance was 0.173, indicating the presence of at least one potentially influential observation according to the 4/n heuristic threshold. This was considered alongside the residual diagnostics and was not used as an automatic exclusion criterion. Examination of the normal P–P plot showed general correspondence with the expected distribution across much of the range, with some departure in the upper tail. The maximum studentized deleted residual was 4.023, indicating the presence of at least one observation with a comparatively large residual. Influence diagnostics, including Cook's distance, were therefore considered alongside the residual distribution when evaluating model performance.

**Table 1:** SAO Workforce and SV by country.

Country	World bank income classification	SAO Workforce per 1,00,000 population	SV per 1,00,000 population	Data adjustment
Albania (Alb)	Upper middle	11.87	2,000.9	Workforce adjusted from 2013 to 2021
Andorra (An)	High	60.27	5,178	Workforce and SV both from 2014
Armenia (Arm)	Upper middle	87.16	5,080	Workforce adjusted from 2017 to 2020
Azerbaijan (Az)	Upper middle	66.86	1,842	Workforce adjusted from 2014 to 2015
Bahrain (Bah)	High	24.44	3,899	Workforce and SV both from 2012
Bangladesh	Lower middle	2.99	161	Workforce adjusted from 2013 to 2011
Belarus (Bls)	Upper middle	125.15	6,945	Workforce adjusted from 2014 to 2020
Belize (Blz)	Upper middle	10.47	1,964	Workforce and SV both from 2014
Bhutan (Bh)	Lower middle	2.69	2,909	Workforce adjusted from 2014 to 2022
Burkina Faso	Lower middle	0.63	328	Workforce adjusted from 2014 to 2012
Cayman Islands (Cay)	High	156.75	8,771	Workforce and SV both from 2015
Chad	Lower middle	0.30	52	Workforce adjusted from 2013 to 2012
China (Chi)	Upper middle	40.13	2,732	Workforce and SV both from 2012
Costa Rica (CR)	Upper middle	22.3	3,746	Workforce and SV both from 2014
Cuba (Cu)	Upper middle	113.14	4,757	Workforce adjusted from 2013 to 2012
Cyprus (Cyp)	High	79.83	3,057	Workforce and SV both from 2014
Ecuador (Ec)	Upper middle	58.55	1,552	Workforce adjusted from 2014 to 2015
Ethiopia (Et)	Low	0.65	43	Workforce adjusted from 2010 to 2011
Fiji (Fi)	Upper middle	5.44	2,247	Workforce adjusted from 2018 to 2020
Georgia (Geor)	Upper middle	130.62	4,473	Workforce adjusted from 2016 to 2020
Guatemala (Gua)	Upper middle	3.03	935.3	Workforce adjusted from 2014 to 2021
Isle of Man (IoM)	High	48.28	9,770	Workforce and SV both from 2014
Israel (Is)	High	77.80	4,879	Workforce adjusted from 2015 to 2014
Jordan (Jo)	Lower middle	24.49	2,982	Workforce and SV both from 2015
Kazakhstan (Kaz)	Upper middle	67.89	3,946	Workforce adjusted from 2015 to 2020
Kiribati (Kir)	Lower middle	8.20	1,718	Workforce and SV both from 2016
Kosovo (Kos)	Upper middle	26.75	1,698	Workforce adjusted from 2014 to 2021
Kyrgyz Republic (Kyr)	Lower middle	43.98	2,677	Workforce adjusted from 2014 to 2020
Moldova (Mol)	Upper middle	93.41	4,279	Workforce adjusted from 2014 to 2020
Mongolia (Mong)	Upper middle	40.10	6,288.6	Workforce adjusted from 2014 to 2020
Morocco	Lower middle	3.73	756	Workforce adjusted from 2016 to 2015
Nauru (Nau)	High	30.00	7,130	Workforce and SV both from 2016
Nepal	Lower middle	2.98	209	Workforce adjusted from 2014 to 2011
New Zealand (Nz)	High	43.00	5,308	Workforce and SV both from 2016
Niger (Nig)	Low	0.41	263	Workforce and SV both from 2014
Oman (Om)	High	29.24	1,609.6	Workforce adjusted from 2017 to 2022
Papua New Guinea (PNG)	Lower middle	2.30	1,264	Workforce and SV both from 2016
Peru (Per)	Upper middle	44.08	3,005	Workforce adjusted from 2014 to 2011
Russian Federation (Rus)	High	62.69	5,439	Workforce adjusted from 2014 to 2020
Samoa (Sam)	Lower middle	1.60	1,552	Workforce and SV both from 2016
San Marino (San)	High	109.79	4,233	Workforce adjusted from 2017 to 2020
Seychelles (Sey)	High	38.60	4,268	Workforce adjusted from 2014 to 2012
Sierra Leone (SL)	Low	0.12	400	Workforce adjusted from 2012 to 2015
Solomon Islands	Lower middle	2.50	868	Workforce and SV both from 2016
South Sudan	Low	0.32	268	Workforce adjusted from 2013 to 2014
Spain (Sp)	High	76.81	6,953.2	Workforce adjusted from 2014 to 2023
Sri Lanka (SriL)	Lower middle	2.78	4,943	Workforce and SV both from 2015
St. Vincent and the Grenadines (sVG)	Upper middle	13.20	2,509	Workforce adjusted from 2014 to 2015
Tajikistan (Taj)	Lower middle	40.67	1,691	Workforce adjusted from 2014 to 2015
Timor-Leste	Lower middle	0.90	433	Workforce and SV both from 2016
Tonga (Tg)	Upper middle	13.98	2,864	Workforce adjusted from 2016 to 2020
Turkmenistan (Tk)	Upper middle	51.45	2,613	Workforce adjusted from 2014 to 2020
Tuvalu (Tv)	Upper middle	18.50	3,417	Workforce and SV both from 2016
Uganda (Ug)	Low	0.97	248	Workforce adjusted from 2012 to 2011
Uzbekistan (Uz)	Lower middle	49.56	2,584	Workforce adjusted from 2014 to 2015
Vanuatu	Lower middle	2.91	860	Workforce adjusted from 2016 to 2020
Yemen, Rep.	Low	0.82	158	Workforce and SV both from 2014

Data from the World Bank with adjustment of SAO according to population growth to the year SV was reported. SAO, Surgeon, Anaesthetic and Obstetric (SAO). SV, surgical volume.

**Figure 1:**
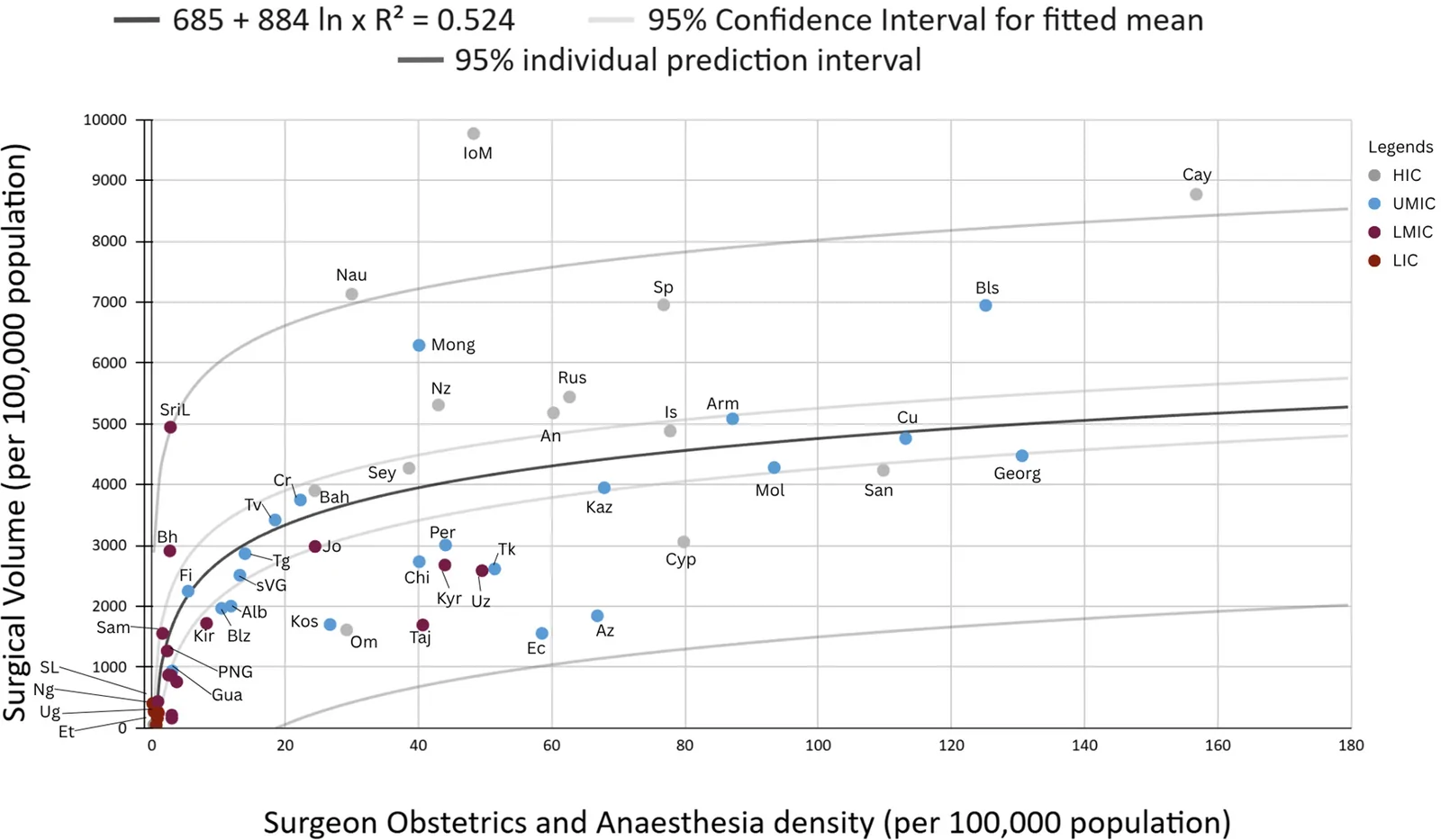
Global Surgical volume according to Surgical density. Both Corresponding years and adjusted Non-corresponding year of reported SAO and Surgical volume plotted. Regression Model y = 884 ln**(x)** + 685 R^2^ = 0.524 High income country(HIC), Upper middle income country(UMIC), Lower middle income country(LMIC), Low income country(LIC). Country labels Albania(Alb), Andorra(An), Armenia(Arm), Azerbaijan(Az), Bahrain(Bah), Belarus(Bls), Belize(Blz), Bhutan(Bh), Cayman Islands(Cay), China(Chi), Costa Rica(CR), Cuba(Cu), Cyprus(Cyp), Ecuador(Ec), Ethiopia(Et), Fiji(Fi), Georgia(Geor), Guatemala(Gua), Isle of Man(IoM), Israel(Is), Jordan(Jo), Kazakhstan(Kaz), Kiribati(Kir), Kosova(Kos), Kyrgyz Republic(Kyr), Moldova(Mol), Mongolia(Mong), Nauru(Nau), New Zealand(Nz), Niger(Nig), Oman(Om), Papua New Guinea(PNG), Peru(Per), Russian Federation(Rus), Samoa(Sam), San Marino(San), Seychelles(Sey), Sierra Leone(SL), Spain(Sp), Sri Lanka(SriL), St Vincent and the Grenadines(sVG), Tajikistan(Taj), Tonga(Tg), Turkmenistan(Tk), Tuvalu(Tv), Uganda(Ug), Uzbekistan(Uz).

The fitted logarithmic equation crosses a predicted SV of zero at an SAO density of approximately 0.46 per 100,000 population. Below this density, the model produces negative fitted SV, which have no physical interpretation. Four countries in the primary dataset (Sierra Leone, South Sudan, Chad and Niger) had SAO densities below this threshold. These fitted values at this extreme lower boundary were therefore not interpreted as literal estimate of SV.

Using the logarithmic regression model, we estimated the change in SV corresponding to the LCoGS surgical workforce density targets of 10, 20, and 40 per 100,000 population. Predicted surgical volumes were presented together with 95% confidence intervals for the fitted mean response generated from the regression model in SPSS. At an SAO density of 10 per 100,000 population, the fitted logarithmic model predicted a surgical volume of 2,720 procedures per 100,000 population. At SAO densities of 20 and 40 per 100,000 population, the corresponding model predicted surgical volumes were 3,333.7 (95% CI for fitted mean: 2,917.2–3,750.2) and 3,946.5 (95% CI for fitted mean: 3,470.7–4,422.3) procedures per 100,000 population, respectively.

The model-implied marginal slope was larger at lower SAO densities and progressively smaller at higher densities, reflecting the mathematical form of the fitted logarithmic relationship. For example, the model-implied marginal slope was approximately 439.4 at an SAO density of 2 per 100,000 population and 87.9 at an SAO density of 10 per 100,000 population [Figure 2].

**Figure 2:**
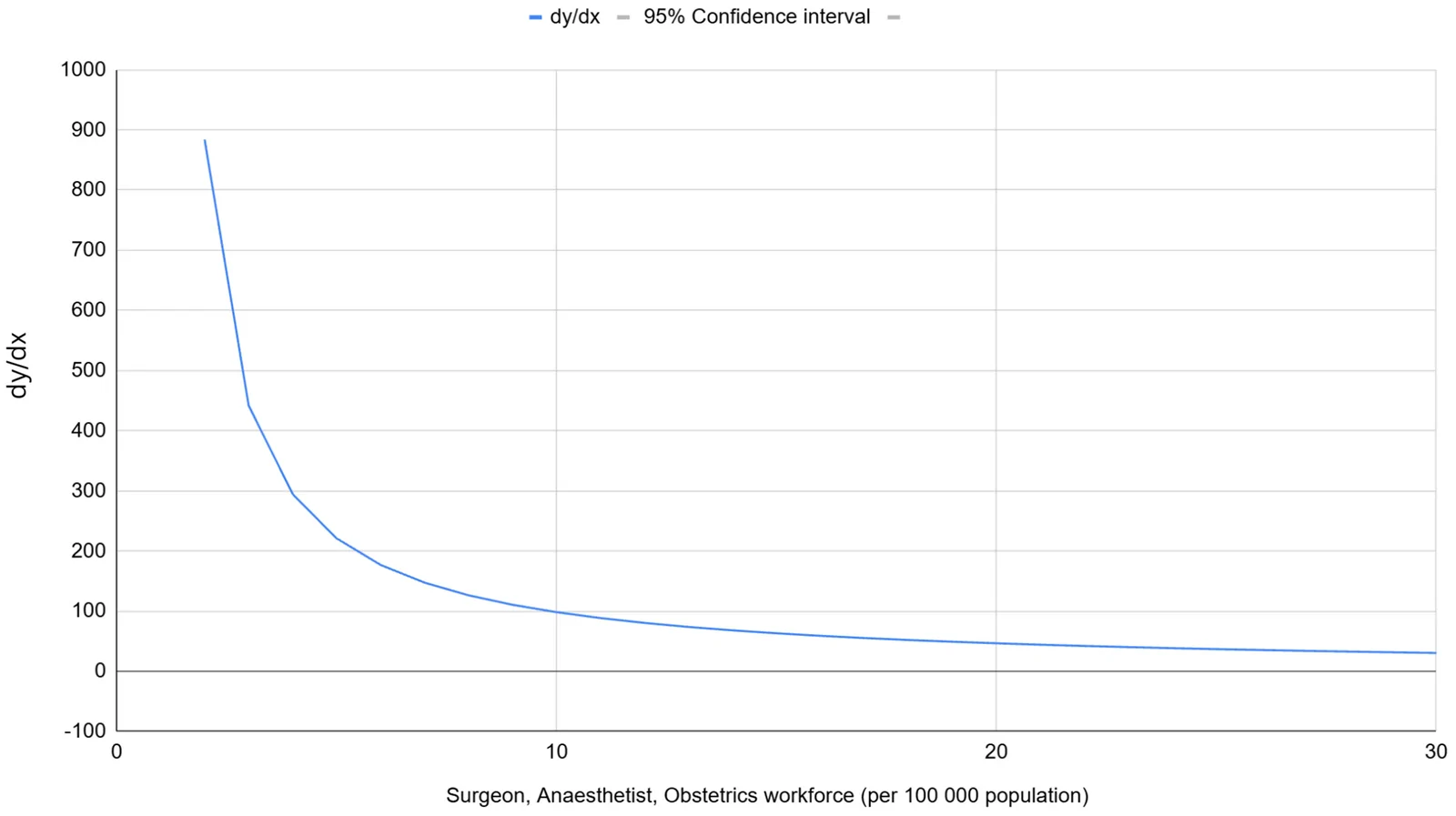
Model implied marginal slope analysis dy/dx = a/x, derived from the primary regression model y = 884 ln**(x)** + 685. The derivative is defined for SAO > 0.

Of the 17 lower-middle-income countries (LMICs), 3 (17.6%) performed above the trendline. Those countries that performed above the trendline were Sri Lanka, Samoa and Bhutan, where Sri Lanka and Bhutan had a higher observed surgical volume than predicted by the fitted cross-country relationship. Of the 21 upper-middle-income countries (UMICs), 6 (28.6%) of them, Fiji, Costa Rica, Mongolia, Armenia, Tuvalu and Belarus, have a higher SV than the predicted SV. Finally, 10 (76.9%) out of 13 HICs performed higher than the predicted model. Within the high-income group, Nauru, Isle of Man and the Cayman Islands outperformed the upper limit of the confidence interval. All low-income countries (LICs) that were included in our model performed below our trendline.

Supplementary Figure S7 presents the residual histograms and normal P–P plots used to assess regression assumptions. Supplementary Table S2 summarises regression statistics and residual/influence diagnostics for the compared models, while Supplementary Table S3 reports temporal-adjustment statistics.

## Discussion

This study found that the association between SV and surgical workforce does not increase linearly, challenging the assumption that expanding the workforce alone will proportionally increase surgical output. The fitted logarithmic model showed a steeper association between SAO density and surgical volume at lower workforce densities, with progressively smaller model-implied increases in surgical volume at higher SAO densities, which highlights previous studies that marginal gains diminish as SAO density increases (12, 13). The fitted relationship was steeper at lower SAO densities and progressively flatter at higher densities. Model predicted surgical volumes at selected SAO densities illustrate this pattern, although these estimates represent cross-country fitted values rather than the effect of adding individual SAO specialists. These diminishing returns highlight the need to reassess how workforce targets translate into service delivery, especially in LMICs where resources are limited and baseline densities are very low (1). However, the result should be interpreted cautiously as this is a correlational study.

Earlier reports echo this finding; in Sierra Leone, doubling the surgical workforce led to only a 15.6% increase in SV, reflecting systemic inefficiencies (12). Similar constraints are observed in HICs such as the United Kingdom, where up to 56% of the surgical workforce reported barriers to operating room access due to workforce burnout, anaesthetic shortages, and infrastructure limitations (14). This illustrates the broader point that workforce capacity must be matched with functional operating environments. Trained staff cannot deliver care without adequate infrastructure, supplies, and support systems (1, 15, 16).

The findings in this study also show that HICs generally had substantial observation relative to the fitted trend supported by earlier work, suggesting that HICs are more effective at improving surgical care than LICs (17). Access to anaesthetic care also remains lower in many LMICs, which is consistent with the generally lower observations than the fitted trend of LMICs observed in our model (18). These findings can be understood through the concept of the “surgical transition”, whereby surgical systems evolve alongside broader economic development, health-system maturity, and changes in disease burden (19). As countries develop, SAO workforce density is expected to rise, and this has been demonstrated in earlier global workforce analyses (1, 3, 4). However, increased workforce density does not necessarily translate proportionally into higher surgical volume, which is consistent with the logarithmic relationship observed in our model. In later stages of surgical transition, health systems tend to provide a greater proportion of complex, resource-intensive, and subspecialised procedures (19). Such operations often require larger multidisciplinary teams, more anaesthetic support, more operating time, and more perioperative resources per case, meaning that additional workforce capacity may be absorbed into increasingly complex care rather than simply increasing the number of procedures performed (1, 19). This provides one possible explanation for the progressively smaller model-implied increases in surgical volume observed at higher SAO densities: as surgical systems mature, growth in workforce density may reflect increasing complexity and quality care as much as rising procedural output.

### Real-world applications

The model provides an alternative representation of the SAO-SV relationship that allows the magnitude of the association to vary across workforce densities, rather than assuming a constant linear relationship. Current global targets, such as the LCoGS recommended 5,000 procedures per 100,000 population, are aspirational but remain largely unattainable for LICs (1). A tiered or incremental approach for SV targets may provide useful intermediate planning milestones for health systems starting from low baseline capacity. For example, an intermediate target such as 10 SAO per 100,000 population could be evaluated alongside complementary measures of infrastructure, anaesthesia capacity, blood supply and financing (1, 15, 16). Although the fitted relationship is steeper at lower SAO densities, this pattern should not be interpreted as demonstrating that workforce expansion has its greatest causal effect in the lowest density setting, particularly at the extreme lower boundary where the logarithmic model produces non-physical predictions.

Pacific Island Countries face distinct challenges due to small populations. For instance, Palau and the Cook Islands have high SAO densities, but only one anaesthetist each, highlighting the limitations of using SAO density alone (20). These countries are examples of how contextualising the indicators would make sense, where they would have more specific challenges to fulfil their needs.

### Driving equity-focused improvements

The analysis also reveals significant inequities between and within income groups. While 76.9% of HICs in our dataset performed had observed SV above the fitted value, all LICs had the observed SV under the fitted value and many substantially. Even among middle-income countries, outputs varied widely. Mongolia, for example, had higher surgical volume than predicted by the fitted model due to strong infrastructure; however, it was pointed out that 80% of their procedures occurred in their capital (21). Furthermore, revised targets must also address the geographic inequity in workforce and service distribution, which reflects the urgent need for spatially disaggregated targets (21). Mexico had a lower observed surgical volume than predicted at 727 procedures per 100,000 population by the fitted model, despite meeting workforce targets. Previous literature has identified health-system fragmentation as one potential contextual factor affecting surgical service delivery in Mexico; however, this was not measured in the present analysis (22).

These disparities highlight the urgent need for equity-focused surgical indicators that reflect geographic access, facility readiness, and socioeconomic barriers, not just national averages. Sri Lanka and Bhutan had higher observed surgical volumes than predicted by the fitted relationship despite constrained resources. The present analysis cannot determine which health-system factors account for these differences, but such observations may identify settings for further contextual investigation.

Surgical service delivery in LMICs faces barriers that align with the LCoGS indicators preparedness, delivery, and financial protection (1, 16). Delays in seeking care are common due to cultural beliefs and financial hardship, leading to reduced SV (12, 16, 23). A major constraint is the shortage and emigration of trained SAO providers, driven by poor working conditions and remuneration (15, 16, 24–28). To address this, task shifting to non-specialist providers has been widely adopted. In a systematic review, non-surgeon physicians and non-physician clinicians performed up to 90% of obstetric surgeries, over a third of general surgery operations, and 43% of non-obstetrics laparotomies (29). While effective in boosting volume, this workforce remains unstable and difficult to track (13).

### Enhancing accountability for surgical system development

The nonlinear representation of the SAO-SV relationship also offers opportunities for increasing accountability in global surgery. It allows stakeholders to monitor whether rising workforce investments are translating into proportional gains in service delivery, and to identify when they are not. For example, Zambia's declining surgical output despite increasing SAO density suggested accountability gaps in the supply chain and system management from past report (30). Moreover, this reinforces the principle that monitoring surgical systems should go beyond input metrics to also include service delivery and outcome performance (12, 25).

### Supporting better planning, financing, and coordination

Refining SAO and SV indicators enhances their role as tools for strategic planning, financing, and international coordination. More nuanced, predictive models help Ministries of Health to better estimate the resource requirements, both human and infrastructural, to meet surgical demand. They also help donors and multilateral organisations align investments with evidence-based needs. The fitted association is steeper at lower workforce densities within the interpretable range of the model, however, this cross-sectional pattern should not be interpreted as demonstrating that early workforce investments are causally more effective. Workforce expansion should instead be considered alongside infrastructure, financing, team capacity and service organisation (28, 31). Such insight can guide global financing instruments and technical support toward where they are most needed.

Financing is therefore a critical mediator between workforce availability and surgical output. In many LMICs, surgical care remains under-prioritised within public budgets and relies heavily on out-of-pocket payments, which can deter patients from seeking care and expose households to catastrophic health expenditure (1, 16, 32). Expanding SAO density without corresponding recurrent funding for operating theatres, anaesthetic services, equipment maintenance, blood products, medicines, referral systems, and workforce retention may therefore produce only limited gains in SV. Sustainable expansion requires predictable domestic funding, risk pooling through tax-funded or social health insurance schemes, and strategic purchasing mechanisms that direct resources towards accessible, efficient, and high-quality surgical services. Performance linked approaches, such as funding complete episodes of care, may also improve how existing surgical capacity is used (31, 32). National Surgical, Obstetric and Anaesthesia Plans should also be fully costed and linked to wider health-system budgets rather than isolated increases in staffing (1, 32). Our model may help guide such financing decisions by showing where workforce investment is likely to be associated with increased surgical volume and where wider financial system barriers may need to be addressed first.

LICs such as Niger, Sierra Leone, and Ethiopia illustrate the importance of this tailored approach. In these settings, task-sharing programs that train non-specialist providers to perform essential surgeries have helped mitigate workforce shortages (15, 24–26). However, persistent infrastructural deficiencies limit the effectiveness of such programs. Reports from Uganda show that more than one-third of surgical facilities operate without essential equipment like oxygen supplies or anaesthesia machines (15). Similarly, in Ethiopia, primary drivers of inter-facility referrals include lack of surgical providers (30%), missing equipment (22%), and absence of blood products (13%), further highlighting the need to align investments with system bottlenecks (25).

In contrast, HICs offer lessons in optimising resource use at later stages of health system development. New Zealand introduced a “package of care” funding model in 2012. This incentivised surgical teams to deliver complete care efficiently per patient episode. This approach not only improved surgical productivity but also reduced reliance on private sector outsourcing (31). Such policy innovations exemplify the value of linking financing to system performance, particularly once basic workforce and infrastructure needs have been met.

### Strengths and limitations

A key strength of this study is its value as a comparative reference point. Countries and facilities with a low surgical workforce yet high SV may provide models of efficiency worth emulating across similar settings. Conversely, settings where an increased workforce does not correspond to higher surgical output, such as in Mexico, Sierra Leone, and Zambia, raise important questions for further investigation (12, 22, 30).

However, the study has several limitations. It does not account for other critical determinants of SV outlined by the WHO, such as infrastructure, governance, financial planning and access barriers, all of which influence surgical productivity. While these factors ultimately impact the observed volume, their exclusion limits the explanatory power. The assumption of uniform productivity across all surgical providers is another limitation. In practice, this varies widely between institutions and countries. For example, in Sierra Leone, differences in workforce efficiency and task distribution significantly affected outcomes (12). Case mix heterogeneity further complicates interpretation, as countries performing a high proportion of minor procedures may appear disproportionately productive. Generally, SV as an indicator is difficult to obtain and is dependent on the method of collection. There is possible underreporting from facilities and also overreporting from household surveys, which is a challenge for institutions to analyse (33).

The role of non-specialist providers is also significant but underrepresented in the current model. In Liberia, non-specialist physicians performed up to 58.3% of surgical procedures, and anaesthesia is often administered by non-physician providers in resource-limited settings (13). Excluding these contributors may underestimate the actual capacity of some health systems. Additionally, the dataset includes extrapolated figures from very small nations (e.g., Andorra, Nauru, Isle of Man), which may distort comparisons and limit generalisability. Differences between facility levels, district vs tertiary hospitals, were not captured, though these have markedly different workforce structures and outputs (13). Additionally, the population adjustment accounts only for changes in the population denominator and assumes that the absolute SAO workforce recruitment remained stable between reporting years. It cannot account for changes in workforce numbers, surgical capacity, infrastructure, financing, case mix or national data collection methods. Differences in how countries measure and report surgical activity may also contribute to the observed variation. Additional limitations include potential selection bias, as only countries reporting both SAO density and surgical volume could be included, and uncertainty arising from temporal harmonisation where indicators were reported in different years. The primary analysis was restricted to countries reporting ≤10,000 surgical procedures per 100,000 population and is therefore subject to range restriction. Estimates from this policy-focused model should not be extrapolated to very high-volume health systems; the unrestricted 67-country analysis is reported separately to demonstrate the influence of these observations on the fitted relationship. Moreover, the data span up to 12 years, which may not reflect current realities. The logarithmic functional form also has a lower bound limitation. Because ln(SAO) decreases without bound as SAO density approaches zero, the fitted equation produces negative SV estimates below approximately 0.46 SAO per 100,000 population. These values are physically impossible and indicate that the model should not be interpreted literally at the extreme low density tail. Model predicted values and model implied marginal slopes in this region should therefore not be used to infer the expected effect of workforce expansion. Finally, unmeasured differences in health-system characteristics may confound the observed cross-country association and limit causal interpretation. Despite these constraints, by incorporating model implied marginal slope analysis and confidence intervals, this study strengthens the reliability of SAO–SV indicators and underscores the importance of context-sensitive metrics. Such refinements can encourage Ministries of Health and policymakers to collect and use surgical data, particularly when low output is clarified as a result of system bottlenecks rather than workforce inefficiency (17).

## Conclusion

This study contributes to the evolving understanding of the relationship between SAO workforce density and surgical volume by demonstrating a nonlinear cross-country association and highlighting the importance of interpreting workforce indicators alongside wider health-system capacity. These findings may help inform future efforts to refine surgical indicators and improve their relevance for equity, accountability and strategic planning, but should be considered exploratory rather than as a validated prediction framework. Future research should build on this work using harmonised longitudinal data, incorporating health-system capacity and other relevant covariates, and externally validating predictive models before their use in policy planning.

## Data Availability

The original contributions presented in the study are included in the article/Supplementary Material, further inquiries can be directed to the corresponding author.

## References

[1] Meara JG Leather AJM Hagander L Alkire BC Alonso N Ameh EA. Global surgery 2030: evidence and solutions for achieving health, welfare, and economic development. Lancet. (2015) 386:569–624. 10.1016/S0140-6736(15)60160-X 25924834

[2] Rose J Weiser TG Hider P Wilson L Gruen R Bickler SW. Estimated need for surgery worldwide based on prevalence of diseases: implications for public health planning of surgical services. Lancet Glob Health. (2015) 3:S13–20. 10.1016/S2214-109X(15)70087-2 25926315 PMC5746187

[3] Holmer H Lantz A Kunjumen T Finlayson S Hoyler M Siyam A. Global distribution of surgeons, anaesthesiologists, and obstetricians. Lancet Glob Health. (2015) 3:S9–11. 10.1016/S2214-109X(14)70349-3 25926323

[4] Holmer H Shrime MG Riesel JN Meara JG Hagander L. Towards closing the gap of the global surgeon, anaesthesiologist, and obstetrician workforce: thresholds and projections towards 2030. Lancet. (2015) 385(Suppl 2):S40. 10.1016/S0140-6736(15)60835-2 26313089

[5] Esquivel MM Molina G Uribe-Leitz T Lipsitz SR Rose J Bickler S. Proposed minimum rates of surgery to support desirable health outcomes: an observational study based on three strategies. World J Surg. (2015) 39:2126–31. 10.1007/s00268-015-3092-7 25968342

[6] Patil P Nathani P Bakker JM Van Duinen AJ Bhushan P Shukla M. Are LMICs achieving the lancet commission global benchmark for surgical volumes? A systematic review. World J Surg. (2023) 47:1930–9. 10.1007/s00268-023-07029-x 37191692 PMC10310578

[7] Henschke N Probyn K Buckley B McIntosh H Sguassero Y Cogo E. The role of non-physician providers of anaesthesia: a systematic review. Br J Anaesth. (2025) 135:886–97. S0007-0912(25)00471-4. 10.1016/j.bja.2025.06.042 40796491 PMC12674024

[8] Falk R Taylor R Kornelsen J Virk R. Surgical task-sharing to non-specialist physicians in low-resource settings globally: a systematic review of the literature. World J Surg. (2020) 44:1368–86. 10.1007/s00268-019-05363-7 31915975

[9] Shaked A Caldero I Durst A. Safety of surgical procedures performed by residents. Arch Surg. (1991) 126:559–60. 10.1001/archsurg.1991.01410290031005 2021334

[10] The World Bank. World Development Indicators. Specialist surgical workforce per 100,000 population. Available online at: https://data.worldbank.org/indicator/SH.MED.SAOP.P5 Accessed 1 September 2025.

[11] The World Bank. World Development Indicators. Number of surgical procedures per 100,000 population. Available online at: https://data.worldbank.org/indicator/SH.SGR.PROC.P5 Accessed 1 September 2025.

[12] Lindheim-Minde B Gjøra A Bakker JM Van Duinen AJ Van Leerdam D Smalle IO. Changes in surgical volume, workforce, and productivity in Sierra Leone between 2012 and 2017. Surgery. (2021) 170:126–33. 10.1016/j.surg.2021.02.043 33785194

[13] Adde HA Van Duinen AJ Sherman LM Andrews BC Salvesen Ø Dunbar NK. A national enumeration of the surgical workforce, its production and disparities in operative productivity in Liberia. World J Surg. (2022) 46:486–96. 10.1007/s00268-021-06379-8 34839375 PMC8803679

[14] Royal College of Surgeons England: UK **s**urgical workforce census report. Available online at: https://www.rcseng.ac.uk/standards-and-research/surgical-workforce-census/ Accessed 1 September 2025.

[15] Albutt K Punchak M Kayima P Namanya DB Shrime MG. Operating volume and surgical case distribution in Uganda’s public sector: a stratified randomised evaluation of nationwide surgical capacity. BMC Health Serv Res. (2019) 19:104. 10.1186/s12913-019-3920-9 30728037 PMC6366061

[16] Ologunde R Maruthappu M Shanmugarahaj K Shalhoub J. Surgical care in low and middle-income countries: burden and barriers. Int J Surg. (2014) 12:858–63. 10.1016/j.ijsu.2014.07.009 25019229

[17] Wurdeman T Menon G Meara JG BC A. A country-level comparison of access to quality surgical and non-surgical healthcare from 1990 to 2016. PLoS One. (2020) 15:e0241669. 10.1371/journal.pone.0241669 33141856 PMC7608906

[18] Vo D Cherian MN Bianchi S Noel L Lundeg G Taqdeer A. Anesthesia capacity in 22 low and middle income countries. J Anesth Clin Res. (2022) 3:207. 10.4172/2155-6148.1000207

[19] Bakker JM Van Duinen AJ Patil P Nathani P Gyedu A Adde HA. Exploring the concept of surgical transition: surgical activity in the light of economic development in Sierra Leone, Liberia, Ghana and India. Front Surg. (2025) 12:1629829. 10.3389/fsurg.2025.1629828 PMC12394221 40896786

[20] Qin RX Zhang G Lim MX Waqainabete I Tudravu J Turagava J. Assessment of essential surgical and anaesthesia care capacity: a cross-sectional study in five Pacific island countries. Lancet Reg Health West Pac. (2023) 39:100830. 10.1016/j.lanwpc.2023.100830 37484709 PMC10362349

[21] Nunez JM Nellermoe J Davis A Ruhnke S Gonchigjav B Bat-Erdene N. Establishing a baseline for surgical care in Mongolia: a situational analysis using the six indicators from the lancet commission on global surgery. BMJ Open. (2022) 12:e051838. 10.1136/bmjopen-2021-051838 35863828 PMC9316021

[22] Pérez-Soto RH Trolle-Silva AM Valdés GAB-R Sánchez-Morales GE Velázquez-Fernández D Medina AR-D. Timely access to essential surgery, surgical workforce, and surgical volume: global surgery indicators in Mexico. Glob Health Sci Pract. (2023) 11:e2100745. 10.9745/GHSP-D-21-00745 PMC9972376 36853648

[23] Grimes CE Bowman KG Dodgion CM Lavy CBD. Systematic review of barriers to surgical care in low-income and middle income countries. World J Surg. (2011) 35:941–50. 10.1007/s00268-011-1010-1 21360305

[24] Sani R Sanoussi S Didier JL Salifou GM Abarchi H. Rural surgery in Niger: a multicentric study in 21 district hospitals. Indian J Surg. (2013) 77:822–6. 10.1007/s12262-013-1015-0 27011464 PMC4775620

[25] Meshesha BR Sibhatu MK Beshir HM Zewude WC Taye DB Getachew EM. Access to surgical care in Ethiopia: a cross-sectional retrospective data review. BMC Health Serv Res. (2022) 22:973. 10.1186/s12913-022-08357-9 35907955 PMC9338639

[26] Loutfi A McLean AP Pickering J. Training general practitioners in surgical and obstetrical emergencies in Ethiopia. Trop Doct. (1995) 1:22–6. 10.1177/00494755950250S110 7879265

[27] LeBrun DG Dhar D Sarkar MI Imran TMT Kazi SN McQueen KAK. Measuring global surgical disparities: a survey of surgical and anaesthesia infrastructure in Bangladesh. World J Surg. (2012) 37:24–31. 10.1007/s00268-012-1806-7 23052803

[28] Comery B Perry WRG Young S Dare A Matalavea B Bissett IP. Delivery of surgical care in Samoa: perspectives on capacity, barriers and opportunities by local providers. ANZ J Surg. (2020) 90:1910–4. 10.1111/ans.15295 31210420

[29] Hoyler M Hagander L Gillies R Riviello R Chu K Bergström S. Surgical care by non-surgeons in low-income and middle-income countries: a systematic review. Lancet. (2015) 385:S42. 10.1016/S0140-6736(15)60837-6 26313091

[30] Cheelo M Brugha R Bijlmakers L Kachimba J McCauley T Gajewski J. Surgical capacity at district hospital in Zambia from 2012 to 2016. World J Surg. (2018) 42:3508–13. 10.1007/s00268-018-4678-7 29785694

[31] Ashton T Bramley D Armstrong D. Improving the productivity of elective surgery through a new package of care. Health Policy. (2012) 108:45–8. 10.1016/j.healthpol.2012.08.007 22917687

[32] Mengistie CT Girma SM Mengistie BT Yigzaw HD Tadesse SZ Estifanos H. Financing surgery in LMICs: building sustainable and equitable systems for universal surgical access—a narrative review. BMC Surg. (2025) 25:516. 10.1186/s12893-025-03204-0 41174633 PMC12579420

[33] Martens JPJ Van Leerdam D Kanneh S Smalle IO Sankoh O Van Duinen AJ. Surgical volume in Sierra Leone: a comparison between population and facility-based data collection. World J Surg. (2025) 49:2523–30. 10.1002/wjs.70050 40817312 PMC12435603

