# Supplementary material for: The correlation between surgical, anaesthetic and obstetric workforce and surgical volume: a modelling study
Source: Front Health Serv. 2026 Oct 8;6:1920090. doi: 10.3389/frhs.2026.1920090 (PMC13687268; doi:10.3389/frhs.2026.1920090)
Supplement: Supplementary file 1 [file Datasheet1.docx]

Supplementary materials.

Representation

The Surgical, Anaesthesia and Obstetrics (SAO) Workforce and the Surgical Volume.

Representation

SAO workforce is reported as per 100,000 population.

Surgical volume is reported as per 100,000 population.

Methodology

This cross-sectional ecological regression study used available reported global data from the World Bank Database on the number of specialist surgeons, anaesthesiologists and obstetrician density per 100,000 population and compared it with the SV per 100,000 population.^10,11^ The data spans over 12 years from 2011.

The latest number of specialist densities and the SV were included if reported in the same year. Countries reporting more than 10,000 surgical procedures per 100,000 population were classified as extreme high-volume observations and excluded from the primary regression model. This fixed operational threshold excluded 10 countries: Australia, Austria, Belgium, Brazil, Colombia, Denmark, Estonia, Finland, Latvia and Sweden. The threshold was transparent and reproducible but was not intended as a formal statistical definition of an outlier. A sensitivity analysis including all 67 countries was conducted to assess whether these exclusions materially affected the regression findings.

The correlation between SAO density and SV was visualised using a scatter plot. The SAO workforce and SV were both reported as per 100,000 population.

Countries with non-corresponding reported years were included after adjusting the SAO workforce according to population growth with the following formula.

SAO in year Y = SAO in year X * ($\frac{population in year x}{population year y}$)

The correlation between the lower and upper SAO indicators with the number of procedures as described in the LCoGS was studied. Several conventional functional forms, including linear, logarithmic, power, exponential and polynomial relationships, were explored. The logarithmic model was used for the main analysis as an interpretable descriptive representation of the observed nonlinear association between SAO workforce density and surgical volume, characterised by progressively smaller model-implied increases in surgical volume at higher SAO densities. The model was intended to describe the association over the observed data range and not to represent a finite saturation point or maximum surgical capacity. To calculate the formula of the regression model, we used the following equation:

y = a·ln(x) + b

Where:

y is the dependent variable, in this case, SV per 100,000 population.

x is the independent variable, in this case, SAO per 100,000 population.

a is the coefficient representing the slope. Which was calculated with the formula =Slope(Volume, ln(x))

b is the y-intercept when ln(x) = 0 or x=1. Which was calculated with the formula =Intercept(Volume, ln(x)).

ln(x) is the natural logarithm of x.

IBM Statistical Package for the Social Sciences(SPSS) version 22 was used for analysis and visualization. A linear regression was fitted with ln(SAO) as the independent variable and surgical volume as the dependent variable. Regression coefficients were estimated by minimising the sum of squared residuals, and model fit was summarised using R-squared (R²) on SPSS. Predicted surgical volumes were calculated from the fitted logarithmic regression model. Uncertainty around the estimated mean relationship was represented using 95% confidence intervals for the fitted mean response. As individual countries were also described relative to the fitted relationship, 95% individual prediction intervals were calculated to account for residual variation between country observations. Both intervals were generated from the regression model in SPSS using a 95% confidence level and the appropriate t distribution with 55 residual degrees of freedom. Regression coefficients were reported together with standard errors, 95% confidence intervals, and p-values. Both intervals were generated from the regression model in SPSS using a 95% confidence level and the appropriate t distribution with 55 residual degrees of freedom. Regression coefficients were reported together with standard errors, 95% confidence intervals, and p-values.

Adjusted SAO density were also analyzed by calculating the mean of the change of the adjustment index calculated, and the distance from the reported year to the targeted adjustment year.

*Regression Diagnostics*

Model assumptions were assessed through examination of standardized residual plots, normal probability plots, and Cook’s distance statistics to identify potential heteroskedasticity and influential observations. The regression diagnostics were summarized for 3 regression models to select the best model for the current study.

Figure S1

Including all countries with corresponding years only

Excluded all countries with non-corresponding years

Regression model with a logarithmic curve y = 1629 ln(x) + 439. R^2^ = 0.244

Figure S2 (Model A)

Included countries with corresponding years of reporting both SAO and Surgical Volume

Restricted to countries reporting ≤10,000 surgical procedures per 100,000 population under the policy-focused analytic threshold.

Regression model with a logarithmic curve y = 1319.4ln(x) + 249.44. R^2^ = 0.548

Grey - high income countries. Blue - Upper middle income countries. Purple - Lower middle income countries. Red - Low income countries.

Figure S3

SAO adjusted according to population growth rate which includes both corresponding years and non-corresponding years.

Regression model with power series, y = 418 x^0.558. R^2^ = 0.708

Figure S4

We included countries with both corresponding and non-corresponding years of reporting, and did not exclude any data points. This included 67 countries as listed in Table 1.

Regression model with logarithmic curve y = 620 + 1594 ln(x). R^2^ = 0.257

Figure S5 (Model B)

Using the same model and dataset [Table 1] in Figure S4, we further limited the vertical axis to 10,000 surgical volume. We included the outliers for this regression analysis to visually understand how the potential outliers affected the regression model in relation to the other countries. Regression model with logarithmic curve y = 620 + 1594 ln(x). R^2^ = 0.257

Figure S6 (Model C)

We included countries with both corresponding and non-corresponding years of reporting. The full dataset included 67 countries. For the primary model, 10 countries reporting more than 10,000 surgical procedures per 100,000 population were excluded using the fixed operational threshold described in the Methods, leaving 57 countries. The model including all 67 countries is presented in Figures S5 and S6.

Regression model with logarithmic curve y = 685 + 884 ln(x). R^2^ = 0.524

SAO 10, Vol 2720

SAO 20, Vol 3333

SAO 40, Vol 3946

Dark Gray - High income

Light Blue - Upper middle income

Dark Purple - Lower middle income

Red - Low income

Figure S7

Histograms and P-P Plot of regression analysis for Model A, Model B and Model C respectively.

Histogram and P-P Plot on the left correspond to the regression model A

Histogram and P-P Plot on the middle correspond to the regression model B

Histogram and P-P Plot on the right correspond to the regression model C

Table S1

To address the mismatch of years, SAO was adjusted to the year of Surgical volume reported with the following formula:

A total of 67 countries were tabulated

SAO in year Y = SAO in year X *(Population in year X/ Population in Year Y)

| Country | World bank income classification | SAO Workforce per 100,000 population | SV per 100,000 population | Data adjustment | Number of years adjusted in years |
| --- | --- | --- | --- | --- | --- |
| Albania | Upper middle | 11.87 | 2000 | Workforce adjusted from 2013 to 2021 | 8 |
| Andorra | High | 60.27 | 5178 | Workforce and SV both from 2014 | 0 |
| Armenia | Upper middle | 87.15 | 5080 | Workforce adjusted from 2017 to 2020 | 3 |
| Australia | High | 45.11 | 10156 | Workforce and SV both from 2016 | 0 |
| Austria | High | 141.15 | 13795 | Workforce and SV both from 2014 | 0 |
| Azerbaijan | Upper middle | 66.86 | 1842 | Workforce adjusted from 2014 to 2015 | 1 |
| Bahrain | High | 24.4 | 3899 | Workforce and SV both from 2012 | 0 |
| Bangladesh | Lower middle | 2.99 | 161 | Workforce adjusted from 2013 to 2011 | 2 |
| Belarus | Upper middle | 125.15 | 6945 | Workforce adjusted from 2014 to 2020 | 6 |
| Belgium | High | 65.61 | 20712 | Workforce adjusted from 2014 to 2013 | 1 |
| Belize | Upper middle | 10.47 | 1964 | Workforce and SV both from 2014 | 0 |
| Bhutan | Lower middle | 2.69 | 2909 | Workforce adjusted from 2014 to 2022 | 8 |
| Brazil | Upper middle | 35.64 | 13940 | Workforce adjusted from 2016 to 2015 | 1 |
| Burkina Faso | Lower middle | 0.63 | 328 | Workforce adjusted from 2014 to 2012 | 2 |
| Cayman Islands | High | 156.75 | 8771 | Workforce and SV both from 2015 | 0 |
| Chad | Lower middle | 0.30 | 52 | Workforce adjusted from 2013 to 2012 | 1 |
| China | Upper middle | 40.13 | 2732 | Workforce and SV both from 2012 | 0 |
| Colombia | Upper middle | 22.71 | 27385 | Workforce and SV both from 2015 | 0 |
| Costa Rica | Upper middle | 22.30 | 3746 | Workforce and SV both from 2014 | 0 |
| Cuba | Upper middle | 113.14 | 4757 | Workforce adjusted from 2013 to 2012 | 1 |
| Cyprus | High | 79.83 | 3057 | Workforce and SV both from 2014 | 0 |
| Denmark | High | 68.49 | 21197 | Workforce adjusted from 2014 to 2015 | 1 |
| Ecuador | Upper middle | 58.55 | 1552 | Workforce adjusted from 2014 to 2015 | 1 |
| Estonia | High | 63.44 | 14832.2 | Workforce adjusted from 2017 to 2022 | 5 |
| Ethiopia | Low | 0.65 | 43 | Workforce adjusted from 2010 to 2011 | 1 |
| Fiji | Upper middle | 5.44 | 2247 | Workforce adjusted from 2018 to 2020 | 2 |
| Finland | High | 75.00 | 15870 | Workforce adjusted from 2014 to 2022 | 8 |
| Georgia | Upper middle | 130.61 | 4473 | Workforce adjusted from 2016 to 2020 | 4 |
| Guatemala | Upper middle | 3.03 | 935.3 | Workforce adjusted from 2014 to 2021 | 7 |
| Isle of Man | High | 48.28 | 9770 | Workforce and SV both from 2014 | 0 |
| Israel | High | 77.80 | 4879 | Workforce adjusted from 2015 to 2014 | 1 |
| Jordan | Lower middle | 24.49 | 2982 | Workforce and SV both from 2015 | 0 |
| Kazakhstan | Upper middle | 67.89 | 3946 | Workforce adjusted from 2015 to 2020 | 5 |
| Kiribati | Lower middle | 8.2 | 1718 | Workforce and SV both from 2016 | 0 |
| Kosovo | Upper middle | 26.75 | 1698 | Workforce adjusted from 2014 to 2021 | 7 |
| Kyrgyz Republic | Lower middle | 43.98 | 2677 | Workforce adjusted from 2014 to 2020 | 6 |
| Latvia | High | 67.00 | 18395 | Workforce adjusted from 2016 to 2015 | 1 |
| Moldova | Upper middle | 93.41 | 4279 | Workforce adjusted from 2014 to 2020 | 6 |
| Mongolia | Upper middle | 40.10 | 6288.6 | Workforce adjusted from 2014 to 2020 | 6 |
| Morocco | Lower middle | 3.73 | 756 | Workforce adjusted from 2016 to 2015 | 1 |
| Nauru | High | 30.00 | 7130 | Workforce and SV both from 2016 | 0 |
| Nepal | Lower middle | 2.98 | 209 | Workforce adjusted from 2014 to 2011 | 3 |
| New Zealand | High | 43.00 | 5308 | Workforce and SV both from 2016 | 0 |
| Niger | Low | 0.41 | 263 | Workforce and SV both from 2014 | 0 |
| Oman | High | 29.24 | 1609.6 | Workforce adjusted from 2017 to 2022 | 5 |
| Papua New Guinea | Lower middle | 2.30 | 1264 | Workforce and SV both from 2016 | 0 |
| Peru | Upper middle | 44.08 | 3005 | Workforce adjusted from 2014 to 2011 | 3 |
| Russian Federation | High | 62.69 | 5439 | Workforce adjusted from 2014 to 2020 | 6 |
| Samoa | Lower middle | 1.60 | 1552 | Workforce and SV both from 2016 | 0 |
| San Marino | High | 109.79 | 4233 | Workforce adjusted from 2017 to 2020 | 3 |
| Seychelles | High | 38.60 | 4268 | Workforce adjusted from 2014 to 2012 | 2 |
| Sierra Leone | Low | 0.12 | 400 | Workforce adjusted from 2012 to 2015 | 3 |
| Solomon Islands | Lower middle | 2.50 | 868 | Workforce and SV both from 2016 | 0 |
| South Sudan | Low | 0.32 | 268 | Workforce adjusted from 2013 to 2014 | 1 |
| Spain | High | 76.81 | 6953.2 | Workforce adjusted from 2014 to 2023 | 9 |
| Sri Lanka | Lower middle | 2.78 | 4943 | Workforce and SV both from 2015 | 0 |
| St. Vincent and the Grenadines | Upper middle | 13.20 | 2509 | Workforce adjusted from 2014 to 2015 | 1 |
| Sweden | High | 105.66 | 14380 | Workforce adjusted from 2015 to 2022 | 7 |
| Tajikistan | Lower middle | 40.67 | 1691 | Workforce adjusted from 2014 to 2015 | 1 |
| Timor-Leste | Lower middle | 0.90 | 433 | Workforce and SV both from 2016 | 0 |
| Tonga | Upper middle | 13.98 | 2864 | Workforce adjusted from 2016 to 2020 | 4 |
| Turkmenistan | Upper middle | 51.45 | 2613 | Workforce adjusted from 2014 to 2020 | 6 |
| Tuvalu | Upper middle | 18.50 | 3417 | Workforce and SV both from 2016 | 0 |
| Uganda | Low | 0.98 | 248 | Workforce adjusted from 2012 to 2011 | 1 |
| Uzbekistan | Lower middle | 49.56 | 2584 | Workforce adjusted from 2014 to 2015 | 1 |
| Vanuatu | Lower middle | 2.91 | 860 | Workforce adjusted from 2016 to 2020 | 4 |
| Yemen, Rep. | Low | 0.82 | 158 | Workforce and SV both from 2014 | 0 |

Table S2

Regression statistics and diagnostics

| Metric | Model A [Figure S2] | Model B [Figure S5] | Model C [Figure S6] |
| --- | --- | --- | --- |
| Sample size | 31 | 67 | 57 |
| Correlation (R) | 0.74 | 0.507 | 0.724 |
| R^2^ | 0.548 | 0.257 | 0.524 |
| Adjusted R^2^ | 0.532 | 0.246 | 0.516 |
| F-statistic | 35.13 | 22.502 | 60.64 |
| p-value | <0.001 | <0.001 | <0.001 |
| Standard error of estimate | 1940 | 5038.48 | 1609.96 |
| Slope (Beta for ln(SAO)) | 1319 | 1593.708 | 884.122 |
| Standard error of Slope | 222.6 | 335.971 | 113.536 |
| 95% confidence interval for slope | 864-1774 | 922.7-2264.7 | 656.6-1111.7 |
| Residual statistics | | | |
| Maximum standardized residual | 2.271 | 4.324 | 3.514 |
| Maximum studentized residual | 2.323 | 4.358 | 3.561 |
| Maximum studentized deleted residual | 2.531 | 5.140 | 4.023 |
| Cook’s distance (Threshold >4/n) | | | |
| Maximum distance | 0.137 (0.129) | 0.149 (0.06) | 0.173 (0.07) |

Table S3 Adjustment statistics

|  | Model B [Figure S5] | Model C [Figure S6] |
| --- | --- | --- |
| Countries requiring adjustment, n (%) | 44 (65.6%) | 37(65%) |
| Mean adjustment index (among adjusted values) | 0.9909 | 0.9909 |
| Median adjustment index | 1.0 | 1.0 |
| Standard deviation | 0.0413 | 0.0438 |
| Minimum adjustment (%) | 0.87 | 0.87 |
| Maximum adjustment (%) | 1.1 | 1.1 |
| Years adjusted (years) | | |
| Mean of adjusted years | 2.33 | 2.32 |
| Median adjusted years | 0 (corresponding years) | 0 (corresponding years) |
| Adjusted range | -3 to 9 years | -3 to 9 years |

Regression model C [Figure S6] was thought to be the most representative of the LMICs for the following reasons:

1. A logarithmic curve was used because the surgical volume will be limited by the number of operating theatres, with maximum staffing.
2. The primary policy-focused analysis restricted the analytic range to countries reporting ≤10,000 surgical procedures per 100,000 population, approximately twice the LCoGS benchmark of 5,000 procedures per 100,000 population. This restriction was not intended to identify statistical outliers. The unrestricted 67-country model was analysed separately to examine the influence of very high-volume health systems on the fitted relationship.
3. This model included the non-corresponding years as well, which increased the data points up to 57 countries and has a good fit.
4. Further analysis of this model included how well the different income groups of countries are performing, which is represented above or below the regression model.
5. The 95% confidence interval was used to represent uncertainty around the fitted mean SAO-SV relationship and was not interpreted as an expected range for individual countries.

Both Model B and C were considered the better regression models and were further analysed for standard error, 95% confidence intervals, p-values, residual statistics, Cook’s distance and P-P plots. The dataset, with removed outliers, has a substantially stronger relationship between workforce density and surgical volume. Showing that the extreme observations weaken the explanatory power of the regression model in Figure S8. Although the standardized residuals for both models showed > 3, the residuals for Model B were higher in comparison to the regression model C. Suggesting the regression model B had multiple extreme residuals that deviated from the model. Cook’s distance for both had moderately influential observations, but extreme residuals are more pronounced in the regression model B [Table S2]. Comparing the histogram and normal P-P Plots, there is a greater deviation from normality in the upper tail for the regression model B. The regression model C shows a more symmetrical distribution around zero, showing a better fit to the regression [Figure S10].

Since 44(65%) countries required adjustments by scaling the SAO density according to populational growth, we further compared both regression model A and model C. These adjustments assume workforce numbers remain stable over short periods and only the population denominator was updated. Comparison of the regression models using corresponding years only (Model A) and adjusted SAO densities (Model C) demonstrated similar associations between workforce density and surgical volume. Model A (n = 31) produced an R² of 0.548, while the adjusted SAO model (Model C, n = 57) produced a comparable R² of 0.524 [Table S2]. Both regression coefficients remained within overlapping confidence intervals. These findings indicate that the population based adjustment of SAO density did not materially affect the observed relationship but allowed inclusion of a larger number of countries in the primary analysis. We also analyzed the adjustments that were made and found the range was between 0.87 and 1.11 (standard deviation of 0.0438) with the mean adjustment index of 0.9909. Some of the SAO were adjusted retrospectively instead of prospective adjustment. This ranged from adjusting back 3 years to 9 years ahead of the reported year. Although the range of adjustments was far, the mean adjustment years were 2.32 years with a median of 0 years (corresponding years) [Table S3].
